# Automated Breast Cancer Diagnosis Based on Machine Learning Algorithms

**DOI:** 10.1155/2019/4253641

**Published:** 2019-11-03

**Authors:** Habib Dhahri, Eslam Al Maghayreh, Awais Mahmood, Wail Elkilani, Mohammed Faisal Nagi

**Affiliations:** ^1^College of Applied Computer Sciences (ACS), Al-Muzahimiyah Branch, King Saud University, Riyadh, Saudi Arabia; ^2^Faculty of Sciences and Technology of Sidi Bouzid, University of Kairouan, Kairouan, Tunisia; ^3^Computer Science Department, Yarmouk University, Irbid, Jordan

## Abstract

There have been several empirical studies addressing breast cancer using machine learning and soft computing techniques. Many claim that their algorithms are faster, easier, or more accurate than others are. This study is based on genetic programming and machine learning algorithms that aim to construct a system to accurately differentiate between benign and malignant breast tumors. The aim of this study was to optimize the learning algorithm. In this context, we applied the genetic programming technique to select the best features and perfect parameter values of the machine learning classifiers. The performance of the proposed method was based on sensitivity, specificity, precision, accuracy, and the roc curves. The present study proves that genetic programming can automatically find the best model by combining feature preprocessing methods and classifier algorithms.

## 1. Introduction

Breast cancer is a prevalent cause of death, and it is the only type of cancer that is widespread among women worldwide [1]. Many imaging techniques have been developed for early detection and treatment of breast cancer and to reduce the number of deaths [2], and many aided breast cancer diagnosis methods have been used to increase the diagnostic accuracy [3, 4].

In the last few decades, several data mining and machine learning techniques have been developed for breast cancer detection and classification [5–7], which can be divided into three main stages: preprocessing, feature extraction, and classification. To facilitate interpretation and analysis, the preprocessing of mammography films helps improve the visibility of peripheral areas and intensity distribution, and several methods have been reported to assist in this process [8, 9].

Feature extraction is an important step in breast cancer detection because it helps discriminate between benign and malignant tumors. After extraction, image properties such as smoothness, coarseness, depth, and regularity are extracted by segmentation [10].

Various transform-based texture analysis techniques are applied to convert the image into a new form using the spatial frequency properties of the pixel intensity variations. The common techniques are wavelet transform [11], fast Fourier transform (FFT) [12], Gabor transforms [13], and singular value decomposition (SVD) [14]. To reduce the dimensionality of the feature representation, principal component analysis (PCA) [15] can be applied. Many works have attempted to automate diagnosis of breast cancer based on machine learning algorithms. For example, Malek et al. [16] proposed a method using the wavelet for features extraction and fuzzy logic for classification. Sun et al. [17] studied the problem by comparing features selection methods, whereas Zheng et al. [18] combined K-means algorithm and a support vector machine (SVM) for breast cancer diagnosis. Several works based on clustering and classification have been conducted [7]. Another approach, introduced by Aličković and Subasi [19], applied a genetic algorithm for feature extraction and rotation forest as a classifier.

Finally, a recent work by Bannaie was conducted [20] based on the dynamic contrast-enhanced magnetic resonance imaging (DCE-MRI) technique to extract relevant information. The contribution of the authors of this paper focuses on the preprocessing stage.

Despite significant efforts, methods described in the literature for breast cancer diagnosis can be considered as semi-automatic methods. Kuhn and Johnson [21] defined the hyperparameters as those parameters that cannot be directly estimated from the data. Typically, some model parameters must be tuned to achieve the desired performance from an algorithm. For instance, the learning rate for training a neural network and the parameter C and sigma parameter of SVMs are specified manually because there is no analytical formula to compute the proper value. Thus, choosing the final tuning parameters of any proposed model has not yet been resolved.

**Figure 1:**
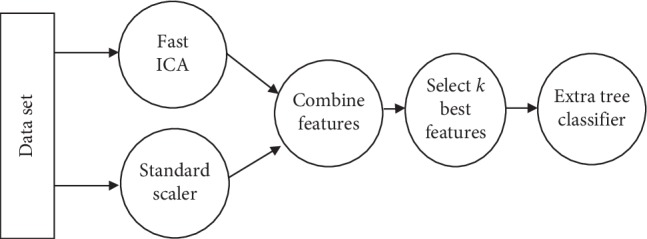
Example of pipeline.

**Figure 2:**
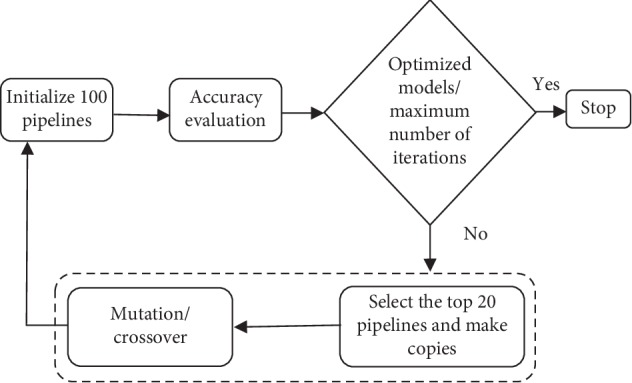
Flowchart of GP.

Nowadays, the demand for machine learning is growing until it becomes a service. Unfortunately, machine learning is still a field with high barriers and often requires expert knowledge. Designing an effective machine learning model including the stages of preprocessing, feature selection, and classification processes requires a set of skills and expertise.  Figure 1 presents an example of a flow of transformations on data called machine learning model or pipeline. At each stage of the pipeline, it is possible to choose different solutions. In the proposed pipeline, the selection of the methods and the parameters in preprocessing process and classification stage is defined automatically. The expert in machine learning chooses the appropriate technique for the current problem domain. However, the nonexperts in machine learning spend a lot of time to optimize their proposed models and to achieve the target performance. In this context, the purpose of the work is to automate the design of the machine learning models using a dozens of techniques. The best combination of the used techniques was optimized by the Genetic programing [22].  Figure 2 depicts the stages of GP algorithm. At each iteration, the pipeline was evaluated according to classification accuracy. To evolve the GP algorithm, the selection, mutation, and crossover operators were applied to find the best pipeline.

In this work, there were two challenges to automate the breast cancer diagnosis: (i) determining which model best fits the data and (ii) how to automatically design and adjust the parameters of the machine learning model.

The remainder of this paper is organized as follows. In Section 2, the materials and methods are explained.  Section 3 summarizes the experimental studies and the obtained results, whereas Section 4 presents the main conclusions.

## 2. Materials and Methods

### 2.1. Dataset Used for Research

In this work, the Wisconsin Breast Cancer dataset was obtained from the UCI Machine Learning Repository. This is the same dataset used by Bennett [23] to detect cancerous and noncancerous tumors. The features were extracted from digitized images of the fine-needle aspirate of a breast mass that describes features of the nucleus of the current image [24]. WDBC database has been effected on 569 patients in Wisconsin hospitals and identified 212 malignant and 357 benign cases. Each observation represents FNA test measurements. For this dataset, the first two attributes correspond to the identifier number and the diagnosis status. The remaining values are the thirty real attributes, including, the mean, the standard error, and the worst of ten cell nucleus features. These ten real values are measured, namely, the radius, texture, perimeter, area, smoothness, compactness, concave points, concavity, symmetry, and fractal dimension.

### 2.2. Related Work

In machine learning, feature selection is the process of choosing a subset of relevant attributes from various candidate subsets, and it is a prerequisite for model building. Feature selection plays a vital role in creating an effective predictive model. There are several benefits to applying the feature selection methods: it (a) is effective and faster in training the machine learning algorithm, (b) reduces the complexity of a model and makes it easier to interpret, (c) improves the accuracy of a model if the right subset is chosen, and (d) reduces overfitting.

Because there may exist a complex interrelation between the features, it is generally difficult to choose the best subset [25]. Different approaches have been proposed in the literature for breast cancer diagnosis [7, 17–20]. Usually, feature selection methods are classified into three general groups: filter, wrapper, and embedded methods [26].

**Figure 3:**
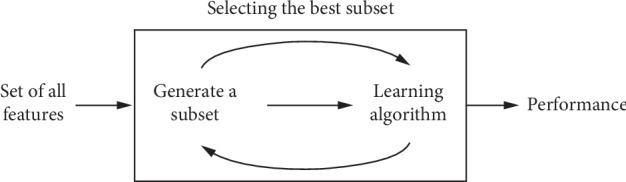
Wrapper methods.

The filter method primarily relies on general features, and it is generally used as a preprocessing step. The subset selection is independent of any specific learning approach. The wrapper approach uses machine learning techniques to choose the optimal subset of features. In other words, the selection of the best features is guided by the learning process, as shown in Figure 3.

The forward feature selection, backward feature elimination, and recursive feature elimination are widely used as wrapper methods.

**Figure 4:**
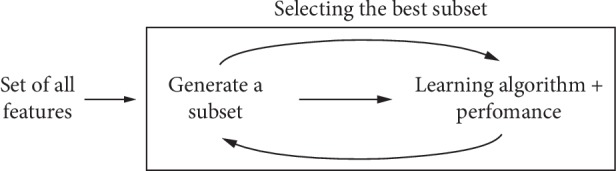
Embedded methods.

Embedded methods combine the qualities of filter and wrapper methods. These are implemented by algorithms that have their own built-in feature selection methods. They perform variable selection as a part of the learning procedure and are usually specific to the given learning machines. The diagram on sequence of data is shown in Figure 4. Wrapper methods were used to conduct the experiments in this study.

### 2.3. The Proposed Method

Pipeline is the process of tying together some ordered final modules into one to build an automated machine learning workflow. It provides high-level abstraction of the machine learning process and significantly simplifies the complete workflow. Mostly, it is known as Extract, Transform, and Load (ETL) operations. Unfortunately, the performance of a machine learning algorithm is determined by number of hyperparameters, including the number of trees in a random forest, the depth, number of hidden layers in the neural network, learning rate, batch size, and degree of regularization.

The purpose of the work is to optimize the list of data transformations and machine learning algorithms to accomplish the classification transformation. To determine the best combination of machine learning algorithm and data is difficult. As a result of the growth of hyperparameter tuning, genetic programming (GP) [22] is proposed to optimize the data and the control parameters of the proposed model. The use of this a well-known evolutionary technique is necessary to find the best combination that leads to highest evaluation results. The GP generates randomly a fixed number of pipelines which constitute the members of the population. Each individual (pipeline) of the population was evaluated based on its fitness which is chosen in this work as the classification score. The implementation of pipelines is based to supervised models from scikit-learn library. The hyperparameters optimized in this work are the number of kennels function for all the classifiers except linear discriminant analysis. The number of kernels function is chosen randomly.

In this work, many applied techniques were tested for the subsequent stages of processing and analysis of the breast cancer dataset.

#### 2.3.1. Stage 1: Preprocessing

As a part of this research, processing was performed on the raw breast cancer data to scale the features using the Standard Scaler module. Standardization of datasets is a common requirement for many machine learning estimators. It transforms the attributes to a standard Gaussian distributions based on (*xi*–mean(*x*))/stdev(*x*) where stdev is the standard deviation. The Robust Scaler depends on the interquartile range to transform the features using (*xi*–*Q*1(*x*))/(*Q*3(*x*)–*Q*1(*x*)), where *Q*1, *Q*2, and *Q*3 represent quartiles. All the transformations used are included in scikit-learn machine learning library [27].

#### 2.3.2. Stage 2: Features Selection

Usually, feature selection is applied as a preprocessing step before the actual learning. However, no algorithm can make good predictions without informative and discriminative features; therefore, to keep the most significant features and reduce the size of the dataset, we implemented PCA using randomized SVD [28].

The module used for feature selection was implemented in using the Python scikit-learn library. All selection strategies were based to many criteria to extract the best features. In our work, feature selection was based on the following modules: removing features with low variance, univariate feature selection, and recursive feature elimination.

#### 2.3.3. Stage 3: Machine Learning Algorithm

Usually, ensemble machine learning algorithms allow better predictive performance compared with a single model. This can be considered machine learning competition, where the winning solution was used as a model for breast cancer diagnosis.

In this paper, the following heterogeneous ensembles machine learning algorithms were used to classify the given data set: support vector machine (SVM) [29], K-nearest neighbor (KNN) [30], decision tree (DT) [31], gradient boosting classifier (GB) [32], random forest (RF) [33], logistic regression (LR) [34], AdaBoost classifier (AB) [35], Gaussian Naive Bayes (GNB) [36], and linear discriminant analysis (LDA) [37].

#### 2.3.4. Stage 4: Parameter Optimization

Genetic Programming (GP) is a type of evolutionary algorithm (EA) that generalizes the genetic algorithm. GP is a model for testing and selecting the best choice among a set of results. Based on biological evolution and its fundamental mechanism (mutation, crossover, and selection), GP generates a solution. The use of GP is the reason for its flexibility; it can model systems where the structure of the desired models and the key features are not known. In this paper, GP allowed the system to search for models from a range of possible model structures and optimizing the pipelines represented in tree structures for the classification problem. GP first generates a fixed number of pipelines based on the primitives described above, such as features selection decomposition. In other words, the sequence of operators evolves to produce machine learning pipelines that are evaluated to maximize the classification accuracy.  Figure 1 depicts an example of a machine learning pipeline. After evaluation of the current pipelines machine learning, a new generation is created based on the highest previous pipelines. Each pipeline is considered an individual of GP. The GP is formed by the three main operators:

  Mutation operator: changing hyperparameters or adding or removing a primitive preprocessing step such as Standard Scaler or the number of trees in a random forest.
  Crossover operator: the crossover operator assumes that 5% of individuals will cross with each other using a 1-point crossover selected at random.
  Selection operator: its main purpose is to select the top 20 individuals and make copies from them. To exchange information between the individuals of the population, the crossover or mutation operator can be applied. The subsequent stages of GP are given in Figure 2.

## 3. Results

In this study, we applied the Wisconsin Breast Cancer dataset to validate the designed models.

According to Breiman et al. [38], a single training and test partitions are not effective estimators of a classification error scheme on a limited dataset. Thus, it was decided that a random subsampling scheme should be used in this experiment to minimize any estimation bias. With the aim of preventing the overfitting, the cross-validation is a powerful concept against this problem. Hence, 10-fold cross-validation was applied to the breast cancer dataset.

As part of the research, three experiments were set up for training of the input data. In the first case, the point of interest was the feature selection stage. In the second experiment, the focal point was the classification model. Finally, the principal focus of the third experiment was automating the previous experiments into one self-regulating process. In other words, the aim was to automate the process of designing and optimizing machine learning algorithms.

In the first experiment, an open-source machine learning software, called WEKA, was employed to extract the features based on the EA, which included (1) particle swarm optimization (PSO) [39], (2) genetic algorithm (GA) [40], (3) evolutionary programming (EP) [22], and a numeric search called best first (BF) [27]. The selected attributes for the previous search methods are shown in Table 1.

Based on the table, it was deduced that the number of features selected for each method was equal to all the tested methods to a certain extent. From the given results of the applied filter features, we understood that only 60% of all the attributes were identical for all the methods, and 80% were identical for the EA. Filter techniques were used to evaluate the pertinence of a feature by looking only at the feature relevance score to remove low-scoring features. Furthermore, all the search methods used required a tuning parameter.

According to Yong et al. [25], there is no single universally optimal feature selection technique, and through various tests conducted, we believe that combining multiple feature extraction methods improved the prediction accuracy of the applied classifiers. We verified that features extraction with hybrid methods improved the performance of the chosen model.

The second experiment compared the popular supervised learning algorithms applied for classification of the problem.

In machine learning algorithms, various metrics are used to evaluate the proposed model.

In this study, the metrics used were accuracy, AUC, confusion matrix, and precision-recall.

Accuracy (ACC) is the measure of correct prediction of the classifier, and it provides general information about how many samples are misclassified. It is defined as

(1) $$\begin{array}{c} \text{ACC}=\frac{\text{TP}+\text{TN}}{\text{FP}+\text{FN}+\text{TP}+\text{TN}}, \end{array}$$

where TP, FP, TN, and FN are the number of true positives, false positives, true negatives, and false negatives, respectively, when the classifier is predicted.

The other metrics derived from a confusion matrix are defined as follows:

(2) $$\begin{array}{c} \text{recall}=\frac{\text{TP}}{\text{TP}+\text{FN}},\\ \text{precision}=\frac{\text{TP}}{\text{TP}+\text{FP}},\\ \text{F}1=2\times \frac{\left(\text{precision}\times \text{recall}\right)}{\left(\text{precision}+\text{recall}\right)}. \end{array}$$

In addition to the previous metrics, receiver operator characteristic (ROC) graphs [41] were employed to represent the relationship between sensitivity (recall) and the specificity metrics. ROC curves represent the performance of a learning algorithm without considering class distribution or error overheads.

The models applied in this experiment were LR, LDA, K-neighbors' classifier, DT classifier, GNB, RF classifier, extra trees classifier, AB, and GB.

**Figure 5:**
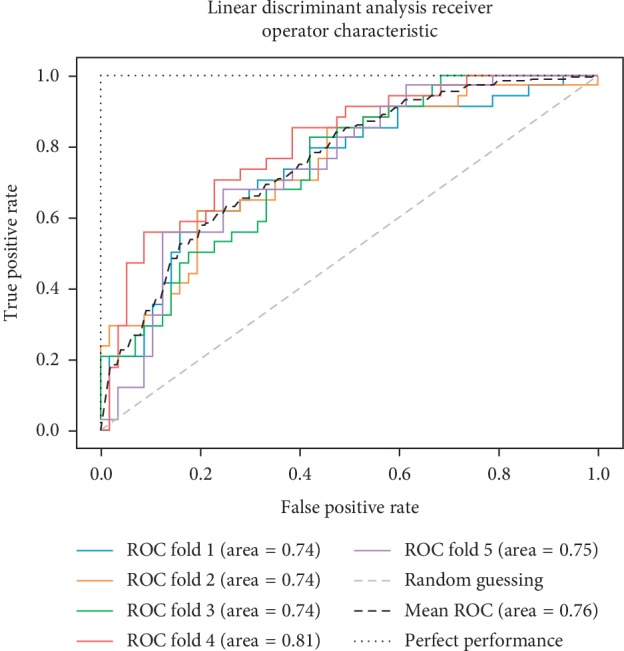
ROC curve for LDA.

**Figure 6:**
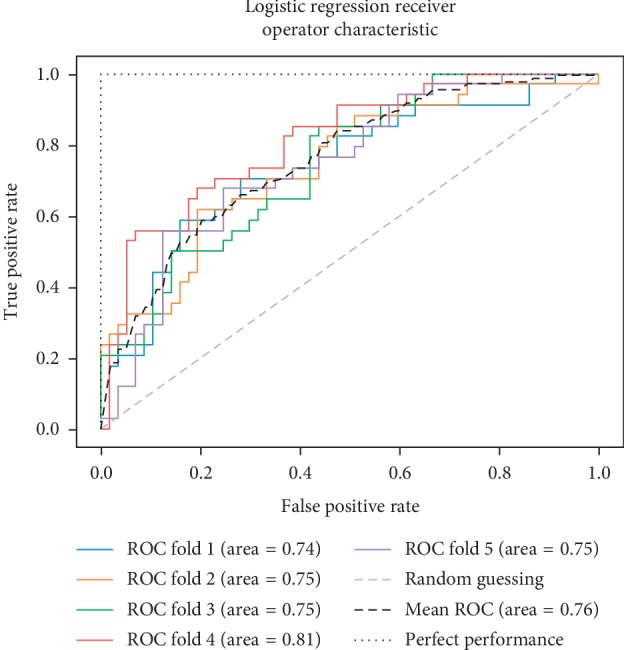
ROC curve for LR.

**Figure 7:**
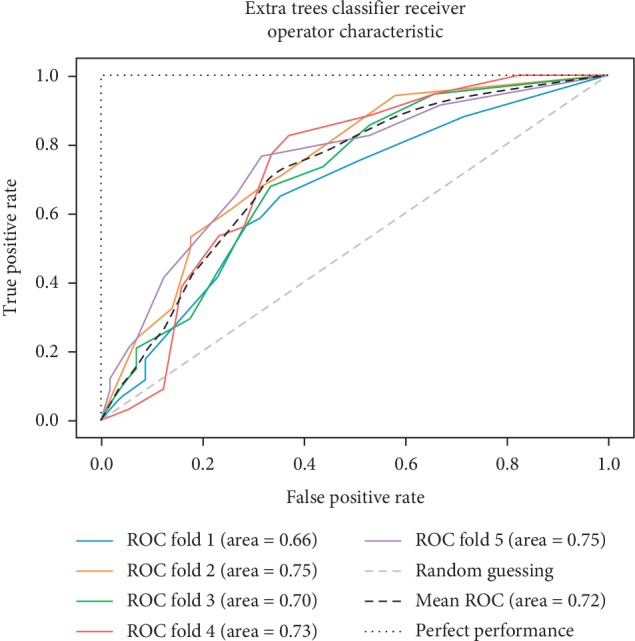
ROC curve for ET.

**Figure 8:**
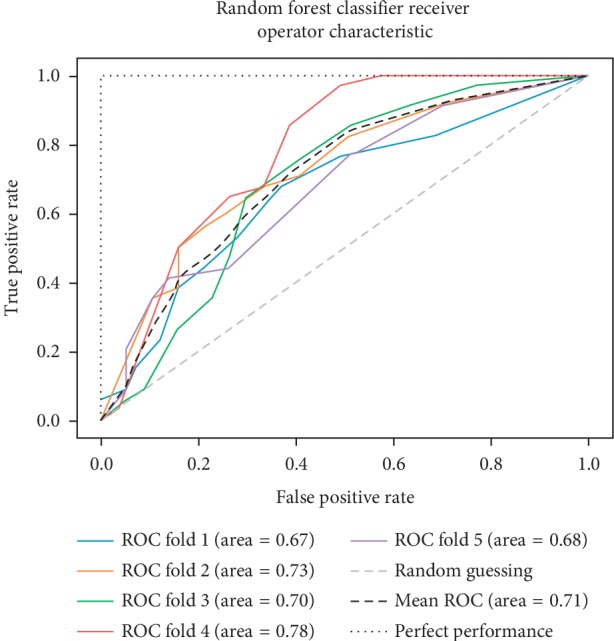
ROC curve for RF.

**Figure 9:**
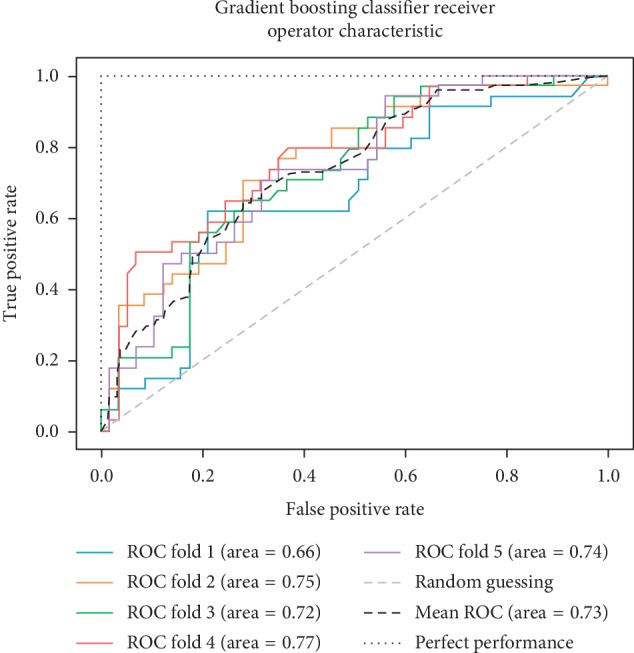
ROC curve for GB.

**Figure 10:**
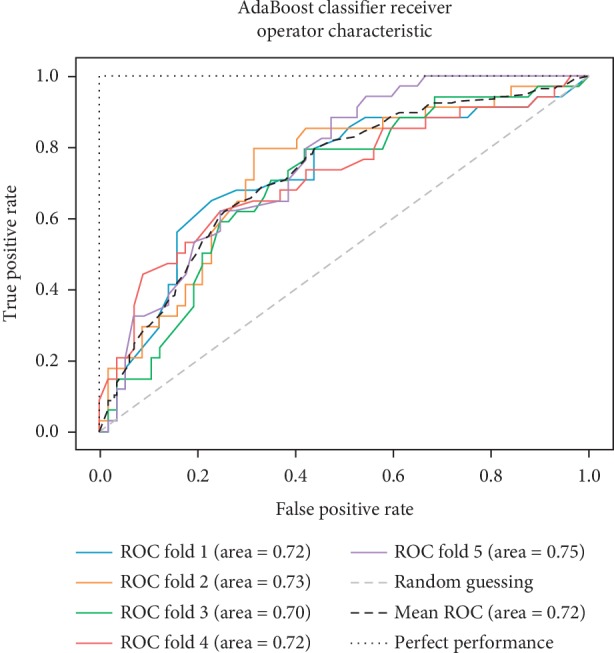
ROC curve for AB.

**Figure 11:**
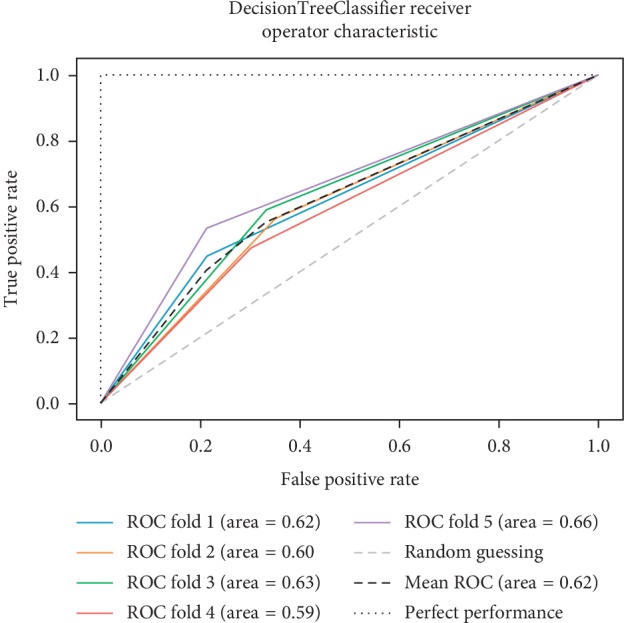
ROC curve for DT.

**Figure 12:**
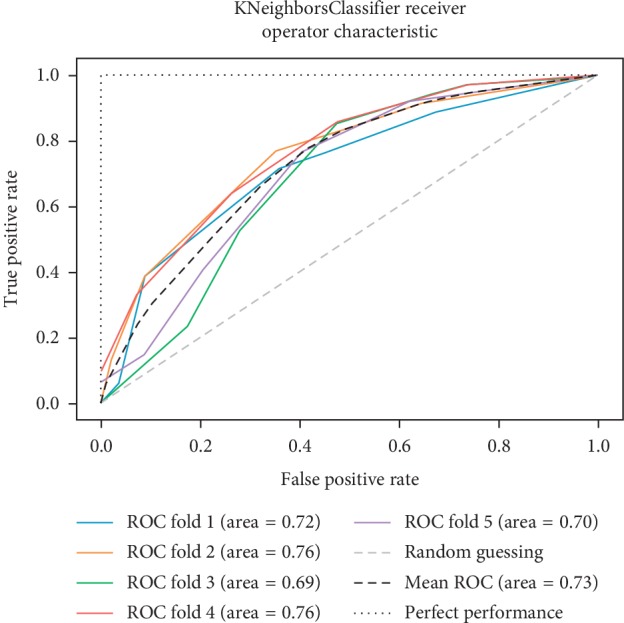
ROC curve for KNN.

**Figure 13:**
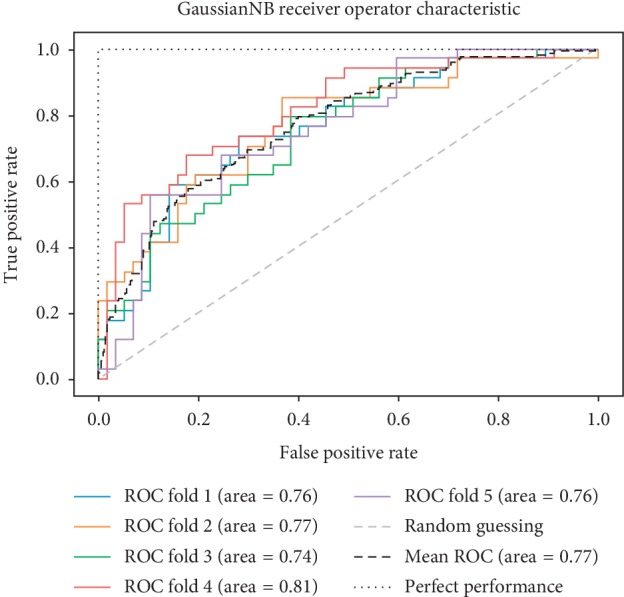
ROC curve for GNB.

As already mentioned, the ROC space is defined with true positives and false positives as the *x* and *y* coordinates, respectively. The ROC curve summarizes the performance across all possible thresholds. The diagonal of the ROC graph can be interpreted as random guessing, and classification models that fall below the diagonal are considered worse than random guessing. A perfect classifier would fall into the top-left corner of the graph with a true positive rate of 1 and a false positive rate of 0. Based on the ROC curve, we can then compute the AUC to characterize the performance of a classification model. Thus, it is shown that applied models can predict more accurately. Figures 5–13 compare the performances of the nine computational models. In our experiment, we found that GNB obtained a higher mean ROC of 77%.

According to Breiman et al. [38], a single train and test partitions are not effective estimators of a classification error scheme. Thus, it was decided that a random subsampling scheme should be used to minimize any estimation bias. Ten-fold cross-validation was used on the breast cancer dataset. In the figures showing the ROC, we applied five-fold cross-validation to obtain clearer figures.

From the previous experiment, we deduced that LR, LDA, and GNB algorithms fit better than the other methods by using the default input parameters for all machine learning classifiers.

## 4. Discussion

Based on the experiments, we prove that combining features selection methods improves the accuracy performance. For this reason, the Genetic programming method was proposed to construct the fixed number of pipelines. Consequently, to automate the process of finding the best pipeline, different machine learning algorithms were applied. Therefore, the proposed approach is considered as one of the solutions to select the proper algorithm and tune hyperparameters, in order to maximize the performance of the model.

The hyperparameters are model selection parameters that are not directly learned within the classifiers, and they control the complexity of the chosen model. There are several possible settings of hyperparameters that need to be carefully determined. In machine learning algorithms, there are no rules to selecting the model parameters. Consequently, many researchers proceed by hand-tuning. In short, the selection of the control parameters affects the prediction performance of the learning algorithm and the model complexity.

The purpose of this work was to address hyperparameter problem. The experiment was divided into three parts. The first experiment focused on comparison of features selection methods by applying the most popular evolutionary algorithms such as PSO and GA. The results of this experiment proved that 80% of the selected features were identical. Usually, most of the methods based on evolution required many control parameters. In many cases, it is difficult to claim expertise with all developed methods, and thus there is a danger of author bias in method selection. To alleviate this bias, we used an optimized control parameter.

**Figure 14:**
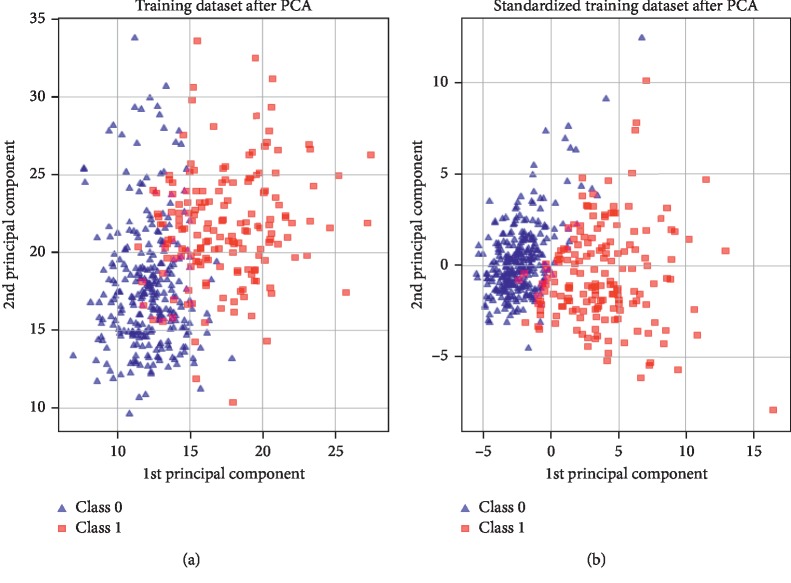
Combining feature extraction.

Through *n* parameters of a given algorithm, the possible configuration will be a hypercube with *n* dimensions. Thus, we thought of applying a simple algorithm for features selection from the breast cancer dataset. Therefore, PCA was chosen for dimensionality reduction. Standardization of the feature selection was justified on the one hand, while on the other hand, the previous algorithms required a few parameters. Moreover, this choice was used by the authors in [42], who presented the advantages of feature extraction and feature selection as (a) preserving the data characteristics for interpretability and (b) resulting in higher discriminating power.  Figure 14 confirms this fact, where the classes are linearly separable after combining PCA and standardization of the feature selection.

The second challenge of the researcher is which machine learning algorithm should be used? Normally, many characteristics need to be considered when choosing any machine learning algorithm, such as accuracy or the complexity. However, many users only consider accuracy. Subsequently, some authors claim that their algorithms perform better than those previously reported do. Usually, most machine learning methods require hyperparameter selection and extensive learning to achieve the best performance.

**Figure 15:**
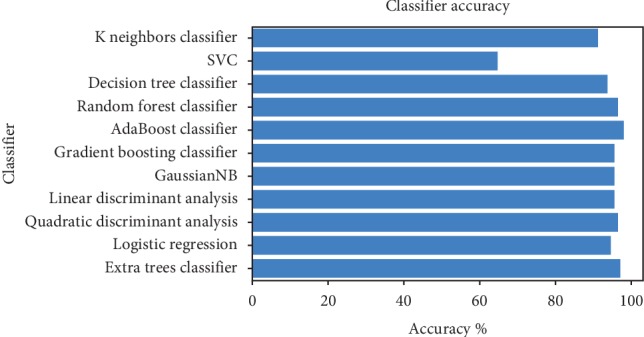
Comparison of classifier accuracy.

**Figure 16:**
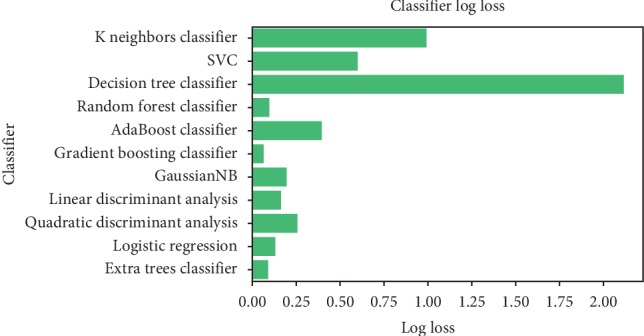
Comparison of log-loss classifier.

**Figure 17:**
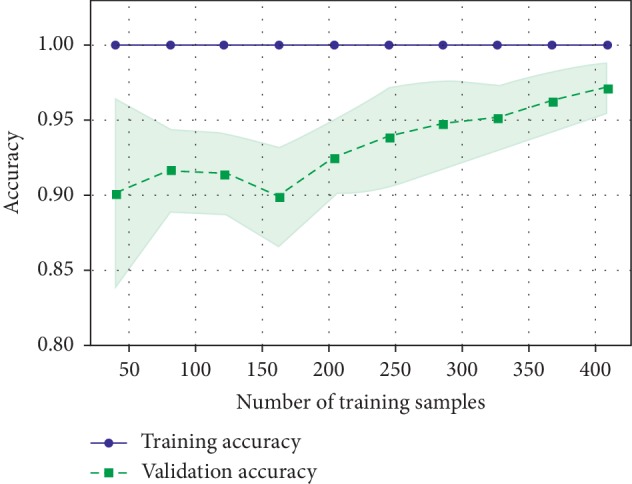
Validation accuracy.

According to the “No Free Lunch” theorem of Wolpert and Macready [43], no algorithm works for every problem. Consequently, many techniques need to be examined for a particular problem before selecting the winner. In this study, we compared the performance of the following machine learning algorithms: KNN, SVM classification, DT, RF, AB, GB, GNB, LDA, quadratic discriminant analysis, LR, and extras classifier. The useful metrics for this experiment were the accuracy and log-loss.  Figure 15 shows the accuracy comparison of useful machine learning, whereas Figure 16 depicts the log-loss. According to Table 2, the AdaBoost classifier seemed to exhibit the best accuracy of 98.24%. However, this is incorrect because the log-loss measure in Table 3 is 0.39 for the AdaBoosting classifier. It is well known that the log-loss allows a more accurate view of the model performance. Owing to the given results, we found three winners: GB classifier, RF classifier, and extra tree classifier. In Figure 17, we plot the variance of the estimate using the standard deviation of the average accuracies. The figure shows a large gap between the training and the cross-validation accuracy curves.

Hence, the accuracy curve justifies the previous results. As mentioned before, the performance of a useful model is sensitive to the control parameters. We therefore tried to automate the full process from features selection to classification. Owing to GP, we built various combinations with the existing modules, as described in Section 2. For each arbitrary-derived building piece, the control parameters were optimized. For example, a random building model can be formed by Standard Scaler module to standardize the input data, recursive feature elimination (RFE) to reduce the features numbers and, finally, the logic regression for classification.

In this work, the control parameters of each chosen algorithm were optimized by the GP techniques. However, human intervention is needed only in initializing the GP parameters such as the population size, generation numbers, and so forth. Owing to the exhaustive list of feature selection techniques and classifier methods, a few algorithms should be chosen to minimize evaluating combinations. The chosen techniques were discussed in the previous section.

In this experiment, we compared the accuracies obtained by the randomly selected methods and we retained the model with high accuracy. After sequential combination of the techniques used, an ensemble of methods was formed. This later model included the MaxAbsSclaer operator for the preprocessing stage, the polynomial features operator to select the appropriate features, and the gradient boosting classifier as the model for supervised classifier. The obtained validation accuracy was 98.24%. The given results demonstrate that combining feature processing and modeling provides a significant improvement without any user intervention.

## 5. Conclusions

This study attempts to solve the problem of automatic detection of breast cancer using a machine learning algorithm. The present algorithm proceeds in different stages. Three different experiments were conducted using the breast cancer dataset.

In the first test, we proved that the three most popular evolutionary algorithms can achieve the same performance after effective configuration. The second experiment focused on the fact that combining features selection methods improves the accuracy performance. Finally, in the last experiment, we deduced how to automatically design the machine learning supervised classifier. Owing to the GP algorithm, we attempted to resolve the hyperparameter problem, which presents a challenge for machine learning algorithms. The proposed algorithm selected the appropriate algorithm from among the various configurations. All experiments were performed using the Python library. Although important results were derived from the proposed method by evaluating an ensemble of approaches from an exhaustive machine learning technique, we encountered a significantly higher time consumption rate. Finally, the proposed model looks naturally suited for control parameter setting of the machine learning algorithms in one side and automated breast cancer diagnosis on the other side.

## Figures and Tables

**Table 1:** Comparison of feature-selection algorithm.

Search algorithm	Number of selected attributes	Numbers
PSO	1, 9, 10, 16, 21, 23, 24, 25, 26, 27, 30, 31	12
Evolutionary search	1, 3, 9, 10, 11, 15, 23, 24, 25, 26, 27, 29, 30	13
Genetic algorithm	1, 7, 9, 10, 16, 21, 23, 24, 25, 26, 29, 30	12
Best first	1, 4, 9, 10, 16, 21, 23, 25, 26, 27, 29, 30	12

**Table 2:** F1-Measurements for breast cancer results.

	GB	DT	RF	GBN	SVM	KNN	AB	LDA	QDA	LR	ET
Benign (%)	96.69	95.36	97.37	96.69	78.72	93.42	98.67	96.73	0.97.26	96.10	98.01
Malignant (%)	93.51	90.91	94.74	93.51	0	86.84	97.44	93.33	95.12	91.89	96.10
Average (%)	95.57	93.80	96.45	95.57	51.10	91.11	98.23	95.33	96.51	94.63	97.34

**Table 3:** Log-loss measure result for breast cancer results.

	GB	DT	RF	GBN	SVM	KNN	AB	LDA	QDA	LR	ET
Log-loss (%)	0.06	2.12	0.09	0.19	0.59	0.992	0.39	0.16	0.25	0.13	0.09

## Data Availability

The data used to support the findings of this study are available from the corresponding author upon request.
